# Chemokines and Cytokines in Immunotherapy of Melanoma and Other Tumors: From Biomarkers to Therapeutic Targets

**DOI:** 10.3390/ijms25126532

**Published:** 2024-06-13

**Authors:** Robin Reschke, Alexander H. Enk, Jessica C. Hassel

**Affiliations:** 1 Department of Dermatology and National Center for Tumor Diseases, University Hospital Heidelberg, 69120 Heidelberg, Germany; 2 German Cancer Consortium (DKTK), DKFZ, Core Center Heidelberg, 69120 Heidelberg, Germany

**Keywords:** chemokines, cytokines, immunotherapy, tumor microenvironment

## Abstract

Chemokines and cytokines represent an emerging field of immunotherapy research. They are responsible for the crosstalk and chemoattraction of immune cells and tumor cells. For instance, CXCL9/10/11 chemoattract effector CD8^+^ T cells to the tumor microenvironment, making an argument for their promising role as biomarkers for a favorable outcome. The cytokine Interleukin-15 (IL-15) can promote the chemokine expression of CXCR3 ligands but also XCL1, contributing to an important DC-T cell interaction. Recruited cytotoxic T cells can be clonally expanded by IL-2. Delivering or inducing these chemokines and cytokines can result in tumor shrinkage and might synergize with immune checkpoint inhibition. In addition, blocking specific chemokine and cytokine receptors such as CCR2, CCR4 or Il-6R can reduce the recruitment of tumor-associated macrophages (TAMs), myeloid-derived suppressor cells (MDSCs) or regulatory T cells (Tregs). Efforts to target these chemokines and cytokines have the potential to personalize cancer immunotherapy further and address patients that are not yet responsive because of immune cell exclusion. Targeting cytokines such as IL-6 and IL-15 is currently being evaluated in clinical trials in combination with immune checkpoint-blocking antibodies for the treatment of metastatic melanoma. The improved overall survival of melanoma patients might outweigh potential risks such as autoimmunity. However, off-target toxicity needs to be elucidated.

## 1. Introduction

Cancer immunotherapies have dramatically improved patient outcomes over the past decade. In particular, checkpoint blockade strategies targeting cytotoxic T-lymphocyte associated protein 4 (CTLA-4) or the programmed cell death protein 1/programmed cell death ligand-1 (PD-1/PD-L1) axis are used in many different cancers including melanoma, non-small lung cancer (NSCLC), renal cell carcinoma, bladder cancer, Hodgkin lymphoma, head and neck cancer, squamous cell carcinoma and Merkel cell carcinoma, among others. For patients with advanced melanoma treated with a combination of anti-PD-1 and anti-CTLA-4 monoclonal antibodies (mAb), the five-year survival rate now exceeds 50% [1]. However, many patients still do not respond clinically, motivating a deeper investigation into the mechanisms of resistance present in non-responders. The baseline and on-treatment frequency of tumor antigen-specific CD8^+^ T cells in the tumor microenvironment and increased levels of interferon-γ (IFN-γ) at baseline are associated with a response to anti-PD-1-therapy in stage III and IV melanoma patients [2,3,4]. The production of IFN-γ by activated T cells contributes to tumor growth control but also induces the additional production of chemokines to recruit additional CD8^+^ T cells into the tumor microenvironment. A higher expression of CCL2, CCL3, CCL4, CXCL9 and CXCL10 transcripts was associated with more CD8^+^ T cell recruitment into melanoma metastases [5]. CXCL9/10/11 are interferon-induced chemokines that recruit effector CD8^+^ and CD4^+^ T cells to the tumor microenvironment by binding them to their cognate receptor CXCR3 [6,7]. In preclinical experiments, Batf3^+^ DCs were identified as the major source of CXCL9 and CXCL10 [8]. Recruitment of the Batf3^+^ DCs themselves also depends on other chemokines, and both CCL4 and XCL1 have been implicated in this process [9,10,11]. Anti-tumor immunity may depend on the intratumoral presence of both Batf3^+^ DCs and tumor-specific CD8^+^ T cells recruited by these chemokines [12]. By understanding the chemokines and cytokines in the tumor microenvironment better, novel immunotherapy targets for patients who do not respond to the existing possibilities for checkpoint blockade could be characterized. In this review, we also discuss other relevant chemokines such as CXCL13, CXCL16, CCL5 and CCL22. Eventually, non-inflamed “cold” tumors could be turned into inflamed “hot tumors” through the local delivery/induction or blockade of essential chemokines. Chemokines can be split up into four groups depending on where their first two cysteine (C) residues are located. The subsets are CC chemokines, CXC chemokines, C chemokines and CX3C chemokines [13]. These chemokines can recruit immune cells to the tumor microenvironment and orchestrate a complex interplay between various cell populations. Targeting cytokines such as IL-4, IL-17, IL-15, IL-2 and IL-6 could be an alternative way to create an inflamed tumor microenvironment. We summarize published in vivo and in vitro melanoma models in which the delivery of chemokines and cytokines into the tumor microenvironment or bloodstream successfully led to tumor shrinkage. Furthermore, we mention examples of currently ongoing clinical studies (phase I/II) that use local or systemic cytokine delivery to treat metastatic melanoma patients.

## 2. CXCL9

C-X-C motif chemokine ligand 9 (CXCL9) is a member of the CXC chemokine family and is induced by interferon-gamma. The signal transducer and activator of transcription 1 (STAT1) and nuclear factor κB (NF-κB) are transcriptional factors which are involved in downstream signaling [14]. They are secreted by various cell types, including immune cells such as dendritic cells or macrophages and non-immune cells like tumor cells and fibroblasts [15,16]. In a melanoma mouse model, Batf3^+^ DCs were a dominant source of CXCL9 [8]. However, tumor-associated macrophages (TAMs) can also express CXCL9 and recruit T cells to the tumor microenvironment [17]. In mice in which the oncogene Src homology 2 domain-containing protein tyrosine phosphatase 2 (Shp2) was knocked out, macrophages produced more of the T-cell chemoattractant CXCL9 [18]. CXCL9 binds to its cognate receptor CXCR3. CXCR3 can be found on activated T lymphocytes and NK cells and on some epithelial cells, as well as cancer cells [19,20]. This binding leads to chemotaxis and proliferation [19]. The upregulation of CXCL9-11 and CCL5 in pretreatment tumors of metastatic melanoma patients were associated with a better overall response to immunotherapy [21,22,23]. In a melanoma mouse model, CD103^+^ DC-derived CXCL9 was required for an effective CD8^+^ T cell response and clinical response to anti-PD-1 therapy [24]. Elevated plasma levels of CXCL9 and CXCL10 a few months after commencing immunotherapy also correlated with this response [24]. When CXCL9 and immunostimulatory factor OX40 ligand were delivered into a colon cancer mouse model via lentivirally transduced mesenchymal stem cells (MSCs), increased numbers of CD8^+^ T and NK cells and an improved efficacy of the anti-PD-1 treatment were observed [25].

## 3. CXCL10

C-X-C motif chemokine ligand 10 (CXCL10) or Interferon gamma-induced protein 10 is also part of the CXC chemokine family [26]. It also binds to CXCR3, a G-protein-coupled receptor. CXCL10 can be induced by IFN-α, -β and -γ [15]. It recruits effector CD4^+^ and CD8^+^ T cells to the tumor microenvironment [8,19,23]. The knockout of CXCR3 in mice resulted in significantly less CD8^+^ T cells infiltrating the tumor [27]. A subsequent anti-PD-1 treatment did not lead to tumor reduction in CXCR3 knockout mice [27]. In preclinical experiments, the major source of CXCL10 within melanomas appeared to be, again, Batf3-lineage DCs, suggesting the key role of these DCs in the anti-tumor T cell response [8]. The public single-cell RNA sequencing data of various metastatic melanoma datasets were re-analyzed and revealed that macrophages were another dominant source of the CXCR3 ligands CXCL9 and 10 following immunotherapy [22]. In addition, not only immune cells but also melanoma cells are able to produce CXCL10 [23]. The response to immunotherapy and immune cell infiltration were correlated with CXCL10 expression in metastatic melanoma patients [22,23], which suggests that CXCL10 is a strong biomarker for a favorable prognosis with immune checkpoint inhibitor (ICI) therapy. Similar results were shown in an in vivo and in vitro melanoma model with an adenovirus vector expressing CXCL10 [28] (Table 1). In colorectal cancer, CXCL10 and CCL5 increased the infiltration of granzyme B^+^ CD8^+^ T cells and IFN-γ^+^ CD4^+^ helper T cells, which enhanced antitumoral activity [29]. In myeloma, the administration of a stabilized CXCL-Ig fusion protein led to reduced tumor growth in mice [30]. Effector CD4^+^, CD8^+^ and NK cells accumulated, whereas the number of Treg cells decreased [30]. A different model of NSCLC-bearing mice also showed reduced tumor growth when the mice were injected with human recombinant CXCL10 [31]. In ovarian cancer cell lines, cyclooxygenase inhibition by indomethacin led to a higher expression of CXCL9 and CXCL10 [32]. The upregulation of CXCL9 and 10 was associated with longer overall survival in patients with high-grade serous ovarian cancer [32]. Interestingly, high CXCL9, CXCL10 and CXCL11 expressions can be observed in patients with immune-related adverse events of the gut and skin who also exhibit a multitude of tissue-resident memory T cells expressing checkpoint molecules such as PD-1, CTLA-4 and LAG-3 [33].

## 4. CXCL13

CXCL13, also known as C-X-C motif chemokine ligand 13 or B-cell-attracting chemokine 1 (BCA-1), binds to the CXCR5 receptor [34]. CXCL13 is a chemokine protein involved in immune cell trafficking and organization within the lymphoid tissues [34]. The transcription factor transforming growth factor beta (TGF-β) can induce CXCL13 production in T cells [35]. A majority of CXCL13 is secreted by follicular helper T cells (Tfh) in the secondary lymphoid organs [34]. It plays a crucial role in the formation of lymphoid follicles by recruiting B cells and T cells to specific areas within lymphoid-like structures. In cancer, the production of CXCL13 also marks the presence of tertiary lymphoid structures (TLSs), which are aggregates of B cells, T cells and DCs, in the tumor microenvironment [36]. CXCL13 and TLS formations in tumors are correlated with the response to immunotherapy in melanoma and other tumors [36,37]. In an ovarian cancer mouse model, CXCL13 in conjunction with anti-PD-1 therapy resulted in tumor infiltration with CXCR5^+^CD8^+^ T cells and improved survival [38], further underscoring the role of CXCL13 as key chemoattractant in ICI-treated cancers.

## 5. CXCL16

C-X-C motif chemokine ligand 16 (CXCL16) binds to the CXCR6 receptor. The CXCR6 receptor is found on CD4^+^, CD8^+^ and Natural Killer T (NKT) cells [39]. In an in vitro immunohistochemistry study, more tumor-infiltrating lymphocytes were recruited by colorectal cancer cells that upregulated CXCL16 [39]. CXCL16 was upregulated in tumor cells in a melanoma mouse model after radiation therapy [40]. CXCR6-deficient mice showed an impaired recruitment of antigen-specific CD8^+^ T cells, in particular of tissue-resident T cells (T_RM_s), in lung tumors [41]. Recruitment of this subset of T cells is highly relevant because T_RM_s are key immune cells for maintaining an anti-tumor equilibrium and mediating ICI-induced responses and toxicity [42,43]. In NSCLC, the CXCL16 expression of cancer and stromal cells was identified as a positive prognostic marker during immunohistochemistry [44]. In CXCR6 knockout mice, the probability of metastases originating from either Lewis lung carcinomas or B16 melanoma cells in the liver was enhanced [45]. Taken together, the CXCL16/CXCR6 axis is important for effector T cell enrichment in the tumor microenvironment of various cancers.

## 6. XCL1

XCL1/lymphotactin binds to XCR1. XCL1 can be produced by CD8^+^ T cells or NK cells [46]. IL-18 induces CXCL16 expression by activating both NF-κB and AP-1 transcription factors [47]. In a melanoma mouse model, the XCL1 and CCL5 produced by NK cells recruited Batf3 DCs to the tumor microenvironment [48]. In humans transcripts of XCL1 and CCL5, these were closely correlated with the signatures for cDC1 and NK cells [48]. Subsequently, the frequency of CXCR3^+^ NK and CXCR3^+^ CD8^+^ T cells was increased. Following an intratumoral injection with Semliki Forest Virus (SFV)-based vectors, the encoding XCL1 and soluble Flt3L (sFlt3L) in mice Batf3 DCs were increased [49]. The intradermal application of a murine XCL1 named mXCL1-V21C/A59C also led to the accumulation of XCR1^+^CD103^+^ DCs [46]. A hepatocellular carcinoma vaccine that combines the XCL1 chemokine and glypican-3 (GPC3) added to the positive effect of anti-PD-1 therapy [50]. XCL1-GPC3 recruited murine XCR1^+^CD8α^+^ DCs and human XCR1^+^CD141^+^ DCs in vitro [50]. The laser-assisted intradermal delivery of a tumor-specific tumor antigen combined with XCL1 resulted in the enhanced priming of CD8^+^ and CD4^+^ effector T cells by dermal DCs and better tumor control in a melanoma mouse model [51]. A fusion protein made of an antigen peptide presented with MHC class I and XCL1 plus an immune adjuvant contributed to reducing tumor growth in a mouse melanoma model in combination with an anti-PD-1 blockade [52].

## 7. CCL2

CC motif ligand 2 (CCL2) is one member of the CC chemokine family and is also called monocyte chemoattractant protein-1 due to its ability to attract and activate macrophages [53]. It can be produced by cancer and stromal cells and binds to the CCR2 receptor. CCL2 can mediate the invasiveness of metastatic melanoma cells [54]. Ligand–receptor interactions lead to the recruitment of protumoral stromal cells as well as myeloid-derived suppressor cells (MDSCs) and tumor-associated macrophages (M2 phenotype) [55,56]. NF-κB and STAT pathways can lead to CCL2 production [57]. In various cancers, higher expression levels of CCL2 are associated with a poor prognosis, such as in breast or liver cancer [58]. In an NSCLC mouse model, the administration of anti-murine-CCL2/CCL12 monoclonal antibodies resulted in reduced tumor growth and increased the influx of CD8^+^ T cells into the tumor [59]. Intratumoral T-regs were reduced. The blockade of CCL2 in combination with an anti-PD-1 blockade in lung cancer-bearing mice enhanced their antitumor efficacy by decreasing the number of MDSCs and increasing the number of CD4^+^ and CD8^+^ T cells and the production of IFN-γ [60].

## 8. CCL4

CC motif ligand 4 (CCL4), also known as macrophage inflammatory protein (MIP)-1β, binds to the CCR5 receptor and belongs to the family of CC chemokines. CCL3 and CCL5 can also bind CCR5. CCL4 can be produced by various immune cells. This includes CD8^+^ T cells but also basophils, which were able to produce CCL4 and contribute to tumor rejection in a mouse melanoma model [61,62]. CCL4 attracts CD103^+^ DCs through the receptor CCR5 [11]. When B-catenin signaling in cold tumors is activated, activating transcription factor 3 (ATF3) is induced and CCL4 transcription is suppressed [11]. This leads to impaired CD103^+^ DC recruitment, which in turn impairs CD8^+^ accumulation via CXCL9/10. A database analysis and immunohistochemistry showed that CCL4 was associated with better overall survival in primary melanoma patients [63]. In a single-cell RNA-sequencing analysis of T cell clones, CCL4 was overexpressed after the immunotherapy of metastatic melanomas [64]. An intravenous infusion of the collagen-binding domain of von Willebrand factor conjugated with CCL4 recruited more CD103^+^ and CD8^+^ T cells into the a melanoma and breast cancer mouse model and improved the immunotherapy’s efficacy [65].

## 9. CCL5

Chemokine (C-C motif) ligand 5 (CCL5) is also known as RANTES (regulated upon activation, normal T cell expressed and secreted) [66]. CCL5 primarily binds to the CCR5 receptor with high affinity. However, it can also bind to other chemokine receptors such as CCR1, CCR3 and CCR4. The complex activation of CCL5 is mediated by IFN, IL-1 and TNF-α through transcription factors, including NF-kB, interferon regulatory factor 1, interferon regulatory factor 3, interferon regulatory factor 7, STAT1 and STAT3 [67]. CCL5 can be produced by lymphocytes, myeloid cells and tumor cells [68]. CCL5 expression was higher in T cell-inflamed melanoma metastases, suggesting its important role in CD8^+^ T cell recruitment into the tumor microenvironment [5]. Similarly, CCL5 transcripts are integrated into the T cell-inflamed gene expression signature, which suggests a benefit for checkpoint therapy [3]. However, CCL5 also recruits TAMs [68]. Anti-CCR5 antibodies lead to the repolarization of TAMs and improved tumor control in colorectal cancer [68]. Together, this suggests that the CCL5/CCR5 axis may have different, and possibly opposing, effects on the immune cell subsets mediating tumor growth and control. Currently, multiple studies seeking to potentiate anti-tumor responses, through CCR5 inhibition, are underway and may serve to better clarify the role of the CCL5/CCR5 axis in tumor control [69].

## 10. CCL22

**Figure 1:**
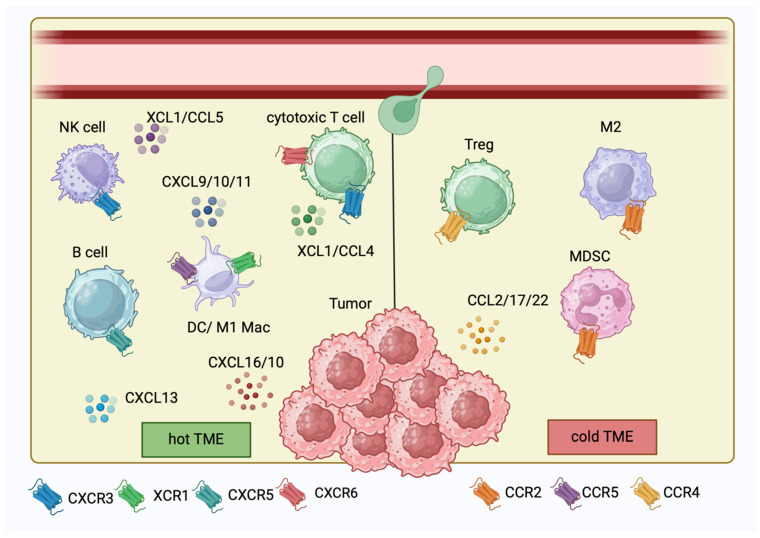
Chemokine network in the tumor microenvironment. Immune cell recruitment from the blood stream via a chemokine gradient and cognate chemokine receptors, shaping a “hot” or “cold” tumor microenvironment (TME), the legend is as follows: chemokine receptors, mac = macrophage, MDSC = myeloid-derived suppressor cell, NK = natural killer, DC = dendritic cell (figure created with biorender).

C-C motif chemokine ligand 22 (CCL22), also known as macrophage-derived chemokine (MDC), is produced by certain tumor cells but also immune cells [70]. The transcription factor PU.1 leads to the expression of CCL22 in dendritic cells and macrophages [71]. It primarily binds to the CCR4 receptor, which is expressed on regulatory T cells (Tregs), Th2-polarized T cells and some subsets of dendritic cells [70]. In the context of cancer, CCL22 has been implicated in promoting tumor progression and immune evasion. Elevated levels of CCL22 have been observed in the tumor microenvironment of melanoma and other tumors [72]. CCL22 plays a role in shaping the immune response within the tumor microenvironment by recruiting immunosuppressive cell populations, such as Tregs. By promoting the accumulation of Foxp3^+^ Tregs, CCL22 and CCL17 contribute to the establishment of an immunosuppressive milieu that facilitates tumors’ immune evasion and progression [73] (Figure 1). CCL22 can be induced by cancer cell-derived IL-1α and the downstream recruitment of Tregs leads to a downregulation of the cell-surface antigen CTLA-4 [74]. Targeting the receptor CCR4 represents a potential therapeutic strategy for cancer treatment. In vitro experiments showed that employing an anti-CCR4 antibody effectively decreased Treg cell counts, facilitating the activation of cancer/testis antigen-specific T cell responses [75]. Blocking CCR4 reduces the infiltration of TAMs and enhances survival in a syngeneic mouse model of pancreatic cancer [76]. CCL22 might serve as a promising biomarker of poor prognosis in cancer [77].

## 11. IL-2

The IL-2 cytokine plays an important role in CD8^+^ T cell immune responses through the stimulation of cell proliferation as well as memory formation [78]. IL-2 is produced predominantly by activated CD4+ T helper cells, though also at lower levels by CD8^+^ T cells, NK cells and activated dendritic cells [79]. IL-2 promotes T cell expansion through binding two receptor classes: high-affinity trimeric receptors containing alpha, beta and gamma chains and low-affinity dimeric receptors containing only the alpha and beta chains [80]. Early uses of recombinant IL-2 in patients with metastatic melanoma led to remarkable anti-tumor responses, though objective responses were limited to 16% of patients [81]. Due to the short half-life of IL-2 of ~7 min, high doses were required to maintain this activity, and many patients developed significant side effects that have limited its clinical applicability [82]. These side effects, characterized as “vascular leak syndrome”, are thought to be, in part, due to expression of the alpha chain of the IL-2 receptor on the vascular endothelium, where it can complete for IL-2 [83]. This differential binding of IL-2 to high- and low-affinity receptors may also explain its limited clinical activity. CD4^+^ Tregs express high-affinity IL-2 receptors and expand in response to IL-2, which may limit the anti-tumor activity of activated CD8^+^ T cells [84]. The intratumoral delivery of IL-2 has also been used, with injected lesions showing increased CD8^+^ T-cell infiltrates, but without significant systemic activity in non-injected lesions [85,86]. Despite its limitations as monotherapy, systemic IL-2 plays an important role in driving the growth and survival of adoptively transferred tumor-infiltrating lymphocytes (TILs). TILs are expanded from resected tumor tissue ex vivo using IL-2, and once a patient has received lympho-depleting chemotherapy, the TILs are re-infused into the patient, followed by a systemic application of high-dose IL-2 [87]. This approach leads to 56% objective responses in treatment-naïve patients with metastatic melanoma, thus highlighting the important role of IL-2 in this promising therapy [88]. In later lines, after anti-PD1 resistance, a cryopreserved TIL product, lifileucel, has demonstrated response rates of still more than 30% and a duration of response not reached after a median follow-up of about 3 years [89]. This treatment has been recently approved by the Food and Drug Administration (FDA) for refractory metastatic melanoma, underscoring the importance of IL-2 in advancing personalized cancer therapy [90]. Unfortunately, a pegylated form of IL-2, bempegaldesleukin, which is thought to preferentially bind to the beta/gamma chains of the IL-2 receptor and expand predominantly CD8^+^ T effector cells, has shown no efficacy in first-line stage IV melanoma. In a randomized phase III study (Pivot IO 001), the combination of nivolumab and bempegaldesleukin revealed even lower response rates compared to nivolumab monotherapy [91]. No significant differences in progression-free and overall survival were seen [91]. In a phase III trial, an intratumoral neoadjuvant application of Darleukin/Fibromun, also called Daromun (L19IL2 + L19TNF) [92], led to improved recurrence-free survival compared to surgery alone in stage III melanoma patients, according to a press release from the company Philogen [93].

Due to the promising results observed in melanoma, Daromun, containing immunocytokines L19IL2 and L19TNF, is also being injected into NMSC tumors (basal cell carcinoma and cutaneous squamous cell carcinoma) as part of the ongoing Duncan trial (Phase II, NCT04362722). Daromun contains a single-chain variable fragment (scFv), called L19, directed against the extra domain B of fibronectin (EDB-FN), which is fused to either IL2 or TNF [94]. EDB-FN is upregulated in tumors experiencing angiogenesis and thereby helps concentrate IL2 in these tumors [94].

## 12. IL-4

IL-4 is a cytokine that plays a central role in regulating immune responses, particularly those involved in allergic reactions, inflammation and the immune response to parasites [95]. It is primarily produced by activated T cells, especially T helper 2 (Th2) cells, mast cells and basophils [95]. IL-4 exerts its effects by binding to its receptors, which are composed of two subunits: IL-4Rα and either the common gamma chain (γc) or the IL-13 receptor alpha 1 (IL-13Rα1) chain [95]. Recently, new evidence has emerged suggesting that IL-4 signaling might have a key role in the efficacy of cancer immunotherapy. In a mouse model of NSCLC IL-4 derived from bone marrow, basophils and eosinophils promoted immunosuppressive myelopoiesis [96]. Based on this finding, a clinical trial of the IL-4Rα blocking antibody dupilumab together with PD-1/PD-L1 ICI, for refractory NSCLC, was initiated [96]. Dupilumab resulted in reduced circulating monocytes, more TILs and a near-complete clinical response in one NSCLC patient [96]. This positive effect of the IL-4 blockade might also be applicable to other solid cancers. The IL-4/STAT6 pathway is involved in colorectal cancer progression [97].

## 13. IL-6

IL-6 is a pleiotropic cytokine produced by multiple cells including macrophages, dendritic cells and endothelial cells [98]. The receptors for IL-6, belonging to the class I cytokine receptor family, require glycoprotein130 (gp130) for signaling and are expressed across numerous different tissues, including hepatocytes and hematopoietic progenitor cells [99]. IL-6 is highly upregulated in severe organ inflammation [100]. In response to infection and injury, IL-6 mediates acute-phase reactants such as C-reactive protein (CRP) and is associated with clinical sequelae such as fever, fatigue, bone loss and stress hormone production [98]. Downstream of gp130 is the Janus kinase (JAK)-STAT3 pathway, which, in multiple pre-clinical models, has been shown to mediate malignant cell growth and promote angiogenesis and metastasis [101]. An IL-6R blockade in conjunction with anti-PD-L1 antibodies in mice implanted with pancreatic cell lines has been shown to improve tumor control and to induce T cell infiltration [102]. Among patients with metastatic melanoma treated with immune checkpoint-blocking antibodies, IL-6 is a significant prognostic factor for survival: elevated baseline and on-treatment levels of IL-6 correlate with worsened overall survival across multiple randomized studies [103]. Based on these observations, the blockade of IL-6 is a promising therapeutic strategy to improve outcomes for patients with advanced melanoma. Tocilizumab, a humanized monoclonal antibody against IL-6R, which had previously been approved for human rheumatologic disorders, will now be tested in combination with immune checkpoint inhibitors in this setting (Table 1).

## 14. IL-12

IL-12 plays a crucial role in bridging innate and adaptive immunity. It is primarily produced by antigen-presenting cells, such as dendritic cells, and it stimulates the differentiation of naive T cells into Th1 cells, promotes the production of IFN-γ and enhances the cytotoxic activity of NK cells and CD8^+^ T cells [104]. For optimal tumor control, the IL-12 produced by Batf3 dendritic cells is essential for effective NK cell function and IFN-γ production [105]. Only Batf3 dendritic cells can produce sufficient IL-12 to achieve an NK cell-mediated control of metastasis [105]. However, the clinical application of IL-12 has been limited by its severe toxicity when administered systemically [104]. To address these challenges, novel strategies have been developed such as targeted delivery systems to maximize IL-12’s therapeutic benefits while minimizing its adverse effects. For example, the antibody-cytokine fusion protein IL12-L19L19 is being tested in melanoma patients in a currently ongoing phase I study (NCT04471987, Table 2). Other promising strategies include local delivery or nanoparticles encapsulating IL-12 [104].

## 15. IL-15

IL-15 belongs to the common γ-chain family of cytokines [106]. IL-15 is a cytokine with proinflammatory properties that binds to the IL-15 alpha receptor chain and the gamma and beta chains of the IL-2 receptor complex [107]. The IL-15 protein is produced by macrophages, monocytes and dendritic cells [108]. It stimulates the proliferation of dendritic cells, T cells and NK cells [109]. IL-15 leads to NK cell development through various downstream signaling pathways, including the Ras-MEK-MAPK, JAK-STAT5 and PI3K-ATK-mTOR pathways [110]. In vivo experiments on melanoma cell lines revealed that IL-15 induces dendritic cells to successfully prime naïve CD8^+^ T cells to differentiate into antigen-specific cytotoxic T cells [111]. The administration of heterodimeric IL-15 into colon cancer-bearing mice led to increased levels of XCL1 as well as the IFN-γ-induced chemokines CXCL9/10 [109]. By using electroporation to deliver plasmid IL-15 into a melanoma mouse model, tumor growth was reduced and survival prolonged [112]. Delivering soluble IL-15 via a Lentivirus resulted in significantly improved survival in 70Z/3-L leukemia-bearing mice [109]. The in vivo half-life and efficacy of IL-15 was improved by generating IL-15/IL-15Rα conjugates [113]. These IL-15 super-agonists show greater potency, bioavailability and stability than soluble IL-15 [114]. They are currently under clinical investigation for patients in combination with immunotherapy agents (Table 2).

## 16. IL-17

Interleukin-17 (IL-17) is a cytokine that plays a significant role in regulating immune responses and inflammation. It is produced primarily by a subset of T cells called T helper 17 (Th17) cells, as well as by other immune cells such as γδ T cells, natural killer T cells and certain subsets of innate lymphoid cells [115]. RAR-related orphan receptor gamma (RORγt) is an IL-17-specific transcription factor [116]. IL-17 is involved in the defense against extracellular pathogens, particularly fungi and bacteria, at mucosal surfaces such as the skin, respiratory tract and gastrointestinal tract [115]. It plays a critical role in coordinating the recruitment of immune cells to sites of infection and in the activation of neutrophils, which are essential for clearing pathogens [115]. In addition to its role in host defense, IL-17 is implicated in the pathogenesis of various inflammatory and autoimmune diseases, including rheumatoid arthritis, psoriasis, multiple sclerosis and inflammatory bowel disease [115]. Dysregulated IL-17 production or signaling can contribute to chronic inflammation and tissue damage in these conditions. Recently, IL-17 expression in the blood and tissue has been described as predicting responses in melanoma patients with a dual checkpoint blockade [117]. High IL-17 levels resulted in an influx of neutrophils and T cells into the tumor microenvironment. Th17 cells can activate Th1 cells and also recruit DCs to the tumor bed and increase the CD8 alpha(+) dendritic cells in tumor-draining lymph nodes [118]. However, in some cancer models, such as breast cancer or lung cancer, increased levels of IL-17 have been described as being pro-tumorigenic and have been associated with poor prognosis [119].

## 17. The Drawbacks of Targeting Chemokines and Cytokines

Chemokines and cytokines also have controversial roles. There is evidence that chemokines have tumor-promoting abilities and effects on autoimmunity as well. CXCL9/10/11 do not only recruit effector CD4^+^ and CD8^+^ to tumor sites but also inflammatory sites in the body. The upregulation of CXCL10 can cause inflammation, such as alopecia areata, or contribute to autoimmunity, e.g., vitiligo [120,121]. Significantly elevated levels of CXCL9 have also been described in autoimmune arthritis [122]. CXCL9 and 10 were also implicated in the development of endocrine autoimmunity such as autoimmune thyroiditis or type 1 diabetes [123]. In addition, CXCL9-11 were significantly upregulated in various ICI-induced immune-related adverse events such as ICI dermatitis and ICI colitis [33,43], demonstrating the potential role of CXCR3 ligands in recruiting T cells to off-target tissue and causing local inflammation [124]. The high levels CXCL9 and 10 produced by endothelial cells can induce the spontaneous migration of melanoma cells and their subsequent metastasis [16]. CXCL16 was implicated in the development of autoimmune encephalomyelitis [125]. In thyroid cancer, CXCL16 was found to increase the infiltration of M2 macrophages and resulted in angiogenesis and a more aggressive cancer phenotype [126]. The CXCL16/CXCR6 axis mediated the improved viability and invasion of lung cancer cell lines in vitro [127]. IL-15 has been described in the pathology of various autoimmune diseases such as rheumatoid arthritis, autoimmune diabetes, inflammatory bowel disease and celiac disease [128]. In a melanoma mouse model, IL-15 promoted tumor progression by stimulating CD215+ myeloid cells [129]. In a B16 melanoma model, the upregulation of CCL4 and CCL5 recruited Tregs in a CCR5-dependent manner and promoted tumor progression [130]. Higher levels of CCL2 and CCL4 in the tumor tissue of lung adenocarcinoma patients predicted unfavorable survival rates [131]. In endometrial carcinoma cells, increased levels of CCL4 meant the upregulation of vascular endothelial growth factor-A (VEGF-A) and tumor growth [132]. CCL5, while mediating CD8^+^ chemoattraction, has also been shown to recruit TAMs and to promote tumor growth by influencing TAM polarization towards a pro-tumor phenotype [68]. There is also contradictory evidence about CCL2 in vivo. In the WM35 melanoma cell line, the CCL2 secreted by tumor cells recruited cytotoxic T lymphocytes by binding to CCR4 [133]. B16 melanoma cells overexpressing CCL2 enhanced Th2 cytokine production and reduced metastatic pulmonary tumor growth in wildtype C57BL/6 mice [134]. IL-2, while supporting CD8^+^ expansion, also acts upon CD4^+^ Tregs, which may in turn limit the net anti-tumor benefit of administering IL-2 alone [84].

## 18. Conclusions and Future Perspectives

A more detailed understanding of which chemokines and cytokines are of relevance for the recruitment of antitumoral effector cells, and in particular CD8^+^ T cells, in human melanoma could open up a therapeutic strategy based on improving immune cell recruitment. If targeting chemokines and cytokines could lead to an influx or potentiation of effector T cells and could turn “cold” tumors into “hot” ones, this might outweigh its potential risks, such as autoimmunity. The effect of chemokine and cytokine deliveries might depend on the stage of the melanoma and on prior treatments with radiation, chemotherapy, targeted therapy or immunotherapy. The delivery of chemokines and cytokines to the tumor microenvironment has already showed their great potential in enhancing the effect of immunotherapy preclinically. It remains to be seen whether targeting these chemokines has a benefit when in combination with anti-PD-1 and anti-CTLA-4 in human metastatic melanoma. Potentially, even patients that did not have a sufficient infiltrate of T cells to mount a strong response to immunotherapy with anti-PD-1 or anti-CTLA-4 could be addressed by administering chemokines and cytokines to the tumor microenvironment locally. The local delivery of chemokines and cytokines could limit the risk of systemic side effects, particularly skin metastases. In the context of metastatic melanoma, anti-IL-15 and anti-IL-6 agents have already made the transitional step into clinical trials (Table 2). Interestingly, in other tumors such as NSCLC, the inhibition of cytokines (here anti- IL-4Rα) is already being tested in combination with an anti-PD1 blockade in the neoadjuvant setting (NCT06088771). The clinical translation of chemokine targets might follow soon. Chemokines and cytokines might also be an interesting avenue for guiding ICI treatment decisions [2]. Using these signatures might be interesting for all melanoma treatment approaches: adjuvant vs. neoadjuvant vs. distant metastases. Currently, promising biomarkers are arising which are based on tumor-intrinsic proteins such as Bax, Bcl-X, PTEN, COX-2, ß-Catenin, MTAP or CD20 for lower stage (I-III) melanomas or late metastases [135,136,137]. However, we are lacking more mechanistic immune-cell-focused biomarkers that indicate a patient’s response to immune checkpoint blockades and guide ICI therapy on an individual immune system-based level at every treatment stage.

**Table 1:** Examples of successful targeting of chemokines and cytokines, sub-categorized into experiments performed on cell lines and/or mice.

	Study	Model	Cell Lines/Mice	Agent
**CXCL10**	[28]	In vitro, in vivo	C57BL⁄6 mice B16F10 melanoma cells	adenovirus vector expressing human CXCL10 (AdCXCL10)
	[138]	In vitro, in vivo	nude mice A375 human melanoma cells	retrovirally transduced A375 human melanoma cells
**CCL2**	[56]	In vitro, in vivo	tumor cell lines NA13, B16F10 and E0771	RS504393, a small-molecule inhibitor of the CCL2 cognate receptor
**CCL4**	[65]	In vitro, in vivo	B16F10 tumor-bearing mice B16F10 melanoma cells EMT6 breast cancer cells	fusion protein of CCL4 and the collagen-binding domain (CBD) of the von Willebrand factor
**XCL1**	[49]	In vivo	C57Bl/6 mice bearing MC38 colon cancer or B16-OVA melanoma	Semliki Forest Virus (SFV)-based vectors encoding XCL1
	[52]	In vivo	C57BL/6J mice B16-OVA cells	fusion protein consisting of an Ag peptide presented with MHC class I, an XCR1 ligand plus an immune adjuvant and polyinosinic:polycytidylic acids (poly(I:C))
	[51]	In vivo	C57BL/6J (B6) mice B16-OVA or B16 melanoma cells	Laser-assisted intradermal delivery of an OVA Ag fused to XCL1
**IL-15**	[112]	In vivo	C57BL/6J mice B16F10 cells	human IL-15 plasmid delivered by electroporation
	[109]	In vitro, in vivo	B6D2F1 mice 70Z/3-L leukemia cells	Lentivirally transduced cells producing soluble IL-15
**IL-2**	[85]	In vivo	Humans with metastatic melanoma	Intratumoral IL-2 +/− chemotherapy
	[86]	In vivo	Humans with metastatic melanoma	Intratumoral IL-2
**IL-6**	[102]	In vivo	KPC-Brca2 mice with Panc02, MT5 or KPC-luc cell lines	Intratumoral anti-IL-6 = anti-PD-L1 antibodies

**Table 2:** Examples of ongoing clinical trials of cytokines in advanced melanoma patients, ^#^ = current enrollment status (30 April 2024), * = stage III/IV melanoma, but also other solid tumors such as renal, lung or bladder cancer, ^+^ = melanoma and other advanced/metastatic solid tumors.

	Study Phase	Patients ^#^	Agent	Cytokine Delivery	ClinicalTrials.gov Identifier
**IL-15**	Phase I/Ib	60	Recombinant heterodimeric IL 5 (IL-15/sIL-15Ra) alone or with Spartalizumab	Subcutaneous	NCT04261439
	Phase IIb	147	Fusion protein of IL-15 mutant bound to IL-15 receptor alpha/immunoglobulin G1 crystallizable fragment (Fc) In combination with Pembrolizumab, Nivolumab, Atezolizumab, Avelumab or Durvalumab	Subcutaneous	NCT03228667
**IL-6**	Phase II	75	Tocilizumab (anti-IL-6R) in combination with nivolumab and ipilimumab	Intravenous	NCT03999749
	Phase II	69	Sarilumab (anti-IL-6R) in combination with ipilimumab, nivolumab and relatlimab	Subcutaneous	NCT05428007
	Phase II	35 *	Tocilizumab (anti-IL-6R) in combination with nivolumab and ipilimumab	Subcutaneous	NCT04940299
**IL-12**	Phase I	94 ^+^	Monoclonal antibody-cytokine fusion protein IL12-L19L19	Intravenous	NCT04471987

## Data Availability

Not applicable.
